# Axial length, more than aging, decreases the thickness and superficial vessel density of retinal nerve fiber layer in non-glaucomatous eyes

**DOI:** 10.1007/s10792-024-02961-w

**Published:** 2024-03-13

**Authors:** Kuan-I. Huang, Fang-Ying Su, Heng-Yen Ho, Heng-Chen Ho, Yan-Wu Chen, Chih-Kuo Lee, Feipei Lai, Henry Horng-Shing Lu, Mei-Lan Ko

**Affiliations:** 1 Department of Ophthalmology, Shin-Kong Wu Ho-Su Memorial Hospital, Taipei, Taiwan; 2 Institute of Statistics, National Chiao Tung University, Hsinchu City, Taiwan; 3 Biotechnology R&D Center, National Taiwan University Hospital Hsinchu Branch, Hsinchu, Taiwan; 4 School of Medicine, National Taiwan University, Taipei, Taiwan; 5 Department of Applied Mathematics, National Sun Yat-Sen University, Kaohsiung, Taiwan; 6 Department of Internal Medicine, National Taiwan University Hospital Hsin-Chu Branch, Hsinchu, Taiwan; 7 Graduate Institute of Biomedical Electronics and Bioinformatics, National Taiwan University, Taipei, Taiwan; 8 Department of Ophthalmology, National Taiwan University Hospital, Hsin Chu Branch, Hsinchu City, Taiwan; 9 Biomedical Engineering and Environmental Sciences, National Tsing Hua University, Hsinchu City, Taiwan

**Keywords:** Axial length, Age, Vessel density, Optical coherence tomography angiography, Retinal nerve fiber layer thickness

## Abstract

**Purpose:**

This study seeks to build a normative database for the vessel density of the superficial retina (SVD) and evaluate how changes and trends in the retinal microvasculature may be influenced by age and axial length (AL) in non-glaucomatous eyes, as measured with optical coherence tomography angiography (OCTA).

**Methods:**

We included 500 eyes of 290 healthy subjects visiting a county hospital. Each participant underwent comprehensive ophthalmological examinations and OCTA to measure the SVD and thickness of the macular and peripapillary areas. To analyze correlations between SVD and age or AL, multivariable linear regression models with generalized estimating equations were applied.

**Results:**

Age was negatively correlated with the SVD of the superior, central, and inferior macular areas and the superior peripapillary area, with a decrease rate of 1.06%, 1.36%, 0.84%, and 0.66% per decade, respectively. However, inferior peripapillary SVD showed no significant correlation with age. AL was negatively correlated with the SVD of the inferior macular area and the superior and inferior peripapillary areas, with coefficients of −0.522%/mm, −0.733%/mm, and −0.664%/mm, respectively. AL was also negatively correlated with the thickness of the retinal nerve fiber layer and inferior ganglion cell complex (*p* = 0.004).

**Conclusion:**

Age and AL were the two main factors affecting changes in SVD. Furthermore, AL, a relative term to represent the degree of myopia, had a greater effect than age and showed a more significant effect on thickness than on SVD. This relationship has important implications because myopia is a significant issue in modern cities.

## Introduction

The pathogenesis of glaucoma has been theorized to correlate with microcirculatory defects in the retina [1, 2]. Since changes in microvasculature may result from or even help cause glaucoma, they may play a key role in the early diagnosis of glaucoma. Optical coherence tomography angiography (OCTA), a novel technology developed in the last decade, provides reproducible and quantifiable information about the microvasculature of the eye without invasive procedures such as injecting special dyes into the veins [3]. OCTA uses intrinsic red blood cells as a contrast agent to build highly accurate three-dimensional models of the microvasculature in designated areas [4, 5]. Nonetheless, OCTA parameters are influenced by several factors, including age, image quality, segmentation errors, degree of myopia, gender, and systemic diseases such as diabetes and hypertensive cardiovascular disease [5–7].

Myopia is a highly prevalent ocular condition worldwide, particularly in East Asian countries [8–11]. For example, the prevalence of myopia in male Taiwanese military conscripts aged 18 to 24 years was 86.1% from 2010 to 2011 [9]. It was predicted by 2050 there will be 4758 million people with myopia (49.8% of the world population) and 938 million people with high myopia (9.8% of the world population) [12]. The rapid consumer electronics growth and the coming “metaverse” may only increase the predicted myopic prevalence instead of decreasing [13]. It is also noteworthy that the secondly leading cause of blindness and moderate and severe vision impairment worldwide was uncorrected refractive error [14]. High myopia also relates to many specific complications such as retinal detachment, glaucoma, cataract, maculopathy, peripapillary deformation, and myopic choroidal neovascularization [15]. A 1-diopter increase in myopia is associated with a 67% increase in the prevalence of myopic maculopathy [16]. That is to say, myopia should be clearly discussed its influences on any parameters. Additionally, eyes with greater degrees of myopia correlate with increased axial length (AL) [17], which may affect the accuracy of OCTA measurements. Interestingly, studies on the relationship between OCTA parameters and AL have been inconsistent. Certain studies have concluded that vessel densities were negatively correlated with axial length [18–20], while other studies have conversely reported that vessel densities were not significantly correlated with axial length [7, 21, 22]. These inconsistencies may be explained by differences in scan areas, race, and prevalence of refraction errors as well as the relatively small populations in those studies.

To rectify such inconsistencies, it is necessary to clarify how particular factors affect OCTA parameters in non-glaucomatous people before it is appropriate to utilize OCTA for glaucoma detection and even for long-term follow-ups. In this study, we began by collecting a relatively large sample size of healthy participants to build a normative reference database of superficial vessel density (SVD) in the macular and peripapillary areas as our control. Next, we analyzed the correlations of SVD with age, AL. The purpose of the present study was to evaluate the interaction of superficial retinal microvasculature changes in non-glaucomatous eyes with AL and age.

## Materials and methods

### Study subjects

This cross-sectional observational study was conducted at the National Taiwan University Hospital Hsin-Chu Branch, Taiwan, and followed the tenets of the Declaration of Helsinki (1964). The participants were recruited into the study when they visited the hospital’s ophthalmologic clinic between June 2019 and February 2020. The protocol was specifically approved by the Institutional Review Board (IRB) of the National Taiwan University Hospital Hsin-Chu Branch (IRB number NTUHHCB 108-025-E). Informed consent was obtained from all participants in the study.

The participants underwent a full ophthalmic examination including refractive error measurement with an ARK-510A (Nidek Co., Gamagori, Japan), slit-lamp examination, gonioscopy, measurement of intraocular pressure (IOP) with an NT-530P Non-Contact Tonometer (Nidek Co., Gamagori, Japan), fundoscopy, color fundus photography with a Visucam 524 (Carl Zeiss Meditec, Inc. Dublin, CA, USA), visual field (VF) testing with a Humphrey Field Analyzer-840 (HFA-840; Carl Zeiss Meditec, Inc. Dublin, CA, USA), and AL measurement with an AL-Scan apparatus (Nidek Co., Gamagori, Japan). The right and left eyes of every subject were evaluated and imaged. The subjects then underwent a blood test at the internal medicine clinic to gather systemic data including serum triglyceride, high-density lipoprotein (HDL), low-density lipoprotein (LDL), serum sugar, glycated hemoglobin (HbA1c), alanine aminotransferase (ALT), creatinine, and uric acid levels; their mean arterial pressure (MAP) and heart rate (HR) were also measured at the internal medicine clinic.

The inclusion criteria for all subjects were an age range of 20 to 80 years, best-corrected visual acuity better than 0.2 (logMAR), no evidence of retinal pathology or glaucoma in either eye, intraocular pressure of 21 mmHg or less, no chronic ocular or systemic corticosteroid use, an open anterior chamber angle on gonioscopy, normal-appearing optic disks (cup–disk ratio less than 0.6 and bilaterally asymmetric cup–disc ratio less than 0.2.), and normal visual field examination results. To avoid poor image quality, an additional inclusion criterion for subjects over 60 years of age was that they had undergone cataract surgery. Exclusion criteria for all eyes included a history of ocular surgery (aside from uncomplicated cataract surgery), ocular trauma, vitreoretinal diseases, signs of diabetic retinopathy, hypertensive retinopathy, maculopathy or optic neuropathy, glaucomatous changes on fundoscopic examination, and signs of unreliable OCTA image quality such as a signal strength index of less than 40. Additionally, we excluded eyes that met the inclusion criteria if glaucomatous change was found in the other eye of the same subject.

### OCTA measurements

All participants underwent OCTA (Angiovue, Optovue Inc., Fremont, CA, USA) examination with a split-spectrum amplitude-decorrelation angiography algorithm. Each OCTA image was manually reviewed to ensure correct segmentation and applicable quality. SVD was measured in all four quadrants of the peripapillary area—superior, inferior, temporal, and nasal—with a scan size of 4.5 × 4.5 mm. The scan depth was between the inner border of the internal limiting membrane and the outer border of the nerve fiber layer (superficial retina). The scan size for the macular area was 3 × 3 mm, and the target regions were two concentric circles whose diameters were 1 mm and 3 mm, both centered on the fovea. The macular area was divided into five sectors: central, nasal, temporal, superior, and inferior. The scan depth was between the inner border of the internal limiting membrane and 10 µm above an outer border of the inner plexiform layer (superficial retina). All images and data were automatically acquired via software built into the measuring apparatus. The images were also used for retinal nerve fiber layer (RNFL) analysis (including RNFL thickness), optic nerve head (ONH) analysis (including cup–disc ratio, rim area, and disc area), and ganglion cell complex (GCC) analysis (including GCC thickness).

### Statistical analysis

Statistical analysis was performed using SAS software version 9.4 (SAS Inc., Cary, NC, USA) and R version 3.6.2. Categorical variables were expressed as percentages. Continuous variables were expressed as the mean, standard deviation, minimum, maximum, and 95% confidence interval. All the participants were divided into three age groups (age < 40 years, 40 years ≤ age < 60 years, age ≥ 60 years) and three AL groups (AL < 24 mm, 24 mm ≤ AL < 26 mm, AL ≥ 26 mm). The average age groups, AL groups and standard deviations were calculated using one-way analysis of variance (ANOVA) followed by Tukey’s test. Pearson’s correlation coefficient was initially used to test the correlation between the outcome variable and continuous variables of age or AL. For further correlations between the outcome variables and all variables, univariate analysis was performed. Pearson’s correlation coefficient was used to test correlations for linear dependence. Two-sample independent *t* tests were used for categorical variables. Multivariable linear regression models with generalized estimating equations (GEE) were used to evaluate associations of the outcome variables, including the SVD of the superior, central, and inferior macula and the superior and inferior peripapillary area, along with the thickness of the superior and inferior RNFL and the superior and inferior GCC, with predictive variables (significant variables in univariate analysis) while adjusting for within-patient and intereye dependence. The working correlation matrix was defined as exchangeable (which indicated compound symmetry), i.e., the measurements from the two eyes were assumed to be equally correlated and independent of the sequence. The plotting functions were implemented in the R packages “rpart” and “rpart.plot.” All tests were two-sided, and a *p* value of < 0.05 was considered significant.

## Results

**Fig. 1:**
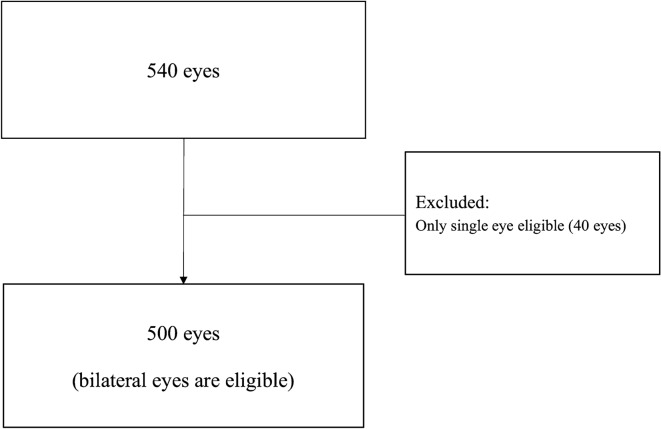
Flow chart illustrating the selection of eligible eyes. After manual evaluation of the raw data to exclude ineligible eyes, 500 eyes were selected for the present study. BCVA = best corrected visual acuity

Of the 1275 eyes from the 661 participants aged 20–80 years who were initially enrolled in the study population, 540 eyes from 290 participants were eventually included in the database of SVD, RNFL thickness and GCC thickness. For further multivariable linear regression models with GEE analysis, we excluded 40 eyes that were selected from those who had only a single eye that fit the inclusion criteria. A flowchart illustrating the selection process based on eligibility criteria is shown in Fig. 1.

Table 1 summarizes the demographics and characteristics of the study population. The mean age was 45.2 ± 14.2 years old, and the patients were divided into three age groups. The under−40 age group made up 37.6% (*n* = 109), the 40–60 age group made up 49% (*n* = 142), and the over−60 age group made up 13.5% (*n* = 39). The systemic data were recorded as the mean ± SD and 95% confidence interval.

**Table 1:** Demographics and characteristics for non-glaucomatous eyes

Total subjects (*N* = 290)	*N* (%)/mean ± SD	95% CI
Age (years)	45.2 ± 14.2	43.5–46.8
< 40	109 (37.5%)	
40–60	142 (49.0%)	
≥ 60	39 (13.5%)	
Sex		
Male	91 (31.4%)	
Female	199 (68.6%)	
SBP (mmHg)	123.8 ± 18.0	121.7–125.9
DBP (mmHg)	73.2 ± 12.3	71.8–74.7
MAP (mmHg)	90.1 ± 13.3	88.6–91.7
HR (/min)	79.3 ± 12.5	77.9–80.8
TG (mg/dL)	106.8 ± 70.3	95.1–118.6
HDL (mg/dL)	56.0 ± 13.8	53.7–58.4
LDL (mg/dL)	113.9 ± 33.1	107.0–120.9
AC sugar (mg/dL)	94.9 ± 14.8	92.5–97.2
HbA1c (%)	6.0 ± 0.7	5.8–6.2
ALT (U/L)	21.5 ± 18.1	18.8–24.3
Creatinine (mg/dL)	0.9 ± 1.2	0.7–1.1
eGFR (mL/min/1.73 m^2^)	99.8 ± 22.2	96.5–103.2
Uric acid (mg/dL)	5.2 ± 1.5	5–5.5

CI = confidence interval; SBP = systolic blood pressure; DBP = diastolic blood pressure; MAP = mean arterial pressure; HR = heart rate; TG = triglyceride; HDL = high-density lipoprotein (cholesterol); LDL = low-density lipoprotein (cholesterol); AC sugar = fasting blood sugar; HbA1c = glycated hemoglobin; ALT = alanine aminotransferase; eGFR = estimated glomerular filtration rate

Ocular data including AL, SVD, IOP, central corneal thickness (CCT), VF in mean defect, cup-to-disc ratio, rim area and disk area are shown in Table 2. The mean AL was 25.2 ± 1.7 mm and was also divided into three groups as follows: (1) 27.4% with AL < 24 mm, (2) 41.1% with AL between 24 mm and 25.9 mm, and (3) 31.5% with AL ≥ 26 mm. The mean SVDs in the superior, central, and inferior macula were 50.3 ± 4.8%, 18.4 ± 6.4%, and 49.7 ± 5.0%, respectively, while the mean SVDs in the superior and inferior peripapillary area were 51.8 ± 4.9% and 52.9 ± 5.2%, respectively. Table 3 shows a trend of significant decreases in the SVD of the macula and the peripapillary areas with increasing age in each age group (all *p* < 0.05). Table 4 also reveals a significant negative correlation between AL and decreasing SVD, RNFL thickness in the superior and inferior quadrants, and GCC thickness in the superior and inferior quadrants among the various AL groups (all *p* < 0.05). Table 5 shows the correlations of OCTA parameters with age or AL as determined by Pearson's correlation test. Significant negative correlations were revealed between age and SVD and all thickness measurements except inferior RNFL thickness. Meanwhile, significant negative correlations were found between AL and the SVD of the inferior quadrant of the macula (*p* < 0.001), both regions of the peripapillary area (*p* < 0.001) and all thickness measurements except superior GCC thickness. Nevertheless, ANOVA and Pearson’s correlation with outcomes not factoring in intereye dependencies might overestimate the strength of associations. Multivariable linear regression models with the GEE were thus adopted; these models are shown in Table 6. Significant negative correlations were found between age and the SVD of the macula (all *p* < 0.001) and superior quadrant of the peripapillary area (*p* < 0.001). According to the β values, the SVD of the superior, central, and inferior macula and superior peripapillary area decreases at rates of 1.06%, 1.36%, 0.84%, and 0.66% per decade, respectively. With regard to AL, significant negative correlations were revealed between AL and SVD of the inferior part of the macula (*p* = 0.011, −0.522%/mm) as well as all peripapillary areas (superior, −0.733%/mm; inferior, −0.664%/mm). On the other hand, a positive correlation was found between AL and SVD of the center part of the macula (+ 1.155%/mm). Superior and inferior RNFL thickness also decreased significantly with longer AL (*p* < 0.001).

**Table 2:** Ocular data for non-glaucomatous eyes

Total eyes (*N* = 540)	*N* (%)/mean ± SD	95% CI
OD/OS		
OD	273 (50.6%)	
OS	267 (49.4%)	
VA (logMAR)	0.1 ± 0.2	0.1–0.12
AL (mm)	25.18 ± 1.71	25.0–25.3
< 24	148 (27.4%)	
24–25.9	222 (41.1%)	
≥ 26	170 (31.5%)	
IOP (mmHg)	14.7 ± 3.4	14.4–15.0
CCT (µm)	544.3 ± 36.5	541.3–547.4
VF: mean defect	−1.33 ± 1.94	-1.69 – -0.97
Macular VD (%)		
Superior	50.3 ± 4.8	49.9–50.7
Center	18.4 ± 6.4	17.9–19.0
Inferior	49.7 ± 5.0	49.3–50.1
Peripapillary VD (%)		
Superior	51.8 ± 4.9	51.4–52.2
Inferior	52.9 ± 5.2	52.4–53.3
RNFL thickness (µm)		
Superior	100.8 ± 9.7	99.9–101.6
Inferior	96.8 ± 9.0	96.1–97.6
GCC thickness (µm)		
Superior	95.9 ± 5.8	95.4–96.4
Inferior	95.3 ± 5.7	94.8–95.8
Cup/disc ratio (%)	49.7 ± 19.5	48.0–51.3
Rim area (× 0.01 mm^2^)	133.3 ± 36.7	130.2–136.4
Disc area (× 0.01 mm^2^)	203.4 ± 48.6	199.3–207.5

CI = confidence interval; OD = right eye; OS = left eye; VA = visual acuity; AL = axial length; IOP = intraocular pressure; CCT = central corneal thickness; VF = visual field; VD = vessel density; RNFL = retinal nerve fiber layer; GCC = ganglion cell complex

**Table 3:** Univariate analysis of OCTA parameters and OCT measurements within age groups

Total eyes (*N* = 540)	Group 1 Age < 40 (*N* = 212)	Group 2 40 ≤ Age < 60 (*N* = 266)	Group 3 Age ≥ 60 (*N* = 62)	*p* value	Tukey’s test
	Mean ± SD	Mean ± SD	Mean ± SD		
Axial length (mm)	25.48 ± 1.55	25.31 ± 1.66	23.63 ± 1.63	< 0.001	1, 2 > 3
Macular VD (%)					
Superior	51.5 ± 3.6	50.1 ± 5.0	46.5 ± 5.4	< 0.001	1 > 2 > 3
Center	20.2 ± 5.7	17.6 ± 6.5	15.4 ± 6.7	< 0.001	1 > 2 > 3
Inferior	50.7 ± 4.2	49.6 ± 5.1	46.4 ± 5.8	< 0.001	1, 2 > 3
Peripapillary VD (%)					
Superior	52.7 ± 4.0	51.5 ± 5.3	49.9 ± 5.3	< 0.001	1 > 2, 3
Inferior	53.5 ± 4.1	52.6 ± 5.7	51.9 ± 6.0	0.043	–
RNFL thickness (µm)					
Superior	101.7 ± 9.8	100.5 ± 9.2	98.5 ± 10.8	0.057	–
Inferior	97.3 ± 8.5	96.7 ± 9.4	95.8 ± 9.1	0.487	–
GCC thickness (µm)					
Superior	95.7 ± 5.2	96.4 ± 6.0	94.3 ± 6.3	0.039	2 > 3
Inferior	95.5 ± 5.1	95.5 ± 6.0	93.8 ± 6.5	0.109	–

VD = vessel density; RNFL = retinal nerve fiber layer; GCC = ganglion cell complex; Tukey's test revealed statistically significant differences in means when comparing groups, with the symbols ' > ' and ' < ' indicating higher or lower means, respectively, in one group compared to another. On the other hand, the symbol ', ' signifies that there were no statistically significant differences between the groups being compared

**Table 4:** Univariate analysis of OCTA parameters and OCT measurement within AL groups

*N* = 540	Group 1 AL < 24 (*N* = 148)	Group 2 24 ≤ AL < 26 (*N* = 222)	Group 3 AL ≥ 26 (*N* = 170)	*p* value	Tukey’s test
	Mean ± SD	Mean ± SD	Mean ± SD		
Age (years)	50.6 ± 16.8	43.0 ± 12.3	40.8 ± 10.9	< 0.001	1 > 2,3
Macular VD (%)					
Superior	49.4 ± 5.0	51.1 ± 3.9	49.9 ± 5.5	0.004	1 < 2
Center	15.4 ± 5.9	18.4 ± 6.3	21.1 ± 5.8	< 0.001	3 > 2 > 1
Inferior	50.0 ± 4.6	50.5 ± 4.2	48.3 ± 5.9	< 0.001	1,2 > 3
Peripapillary VD (%)					
Superior	52.1 ± 4.8	52.7 ± 4.0	50.4 ± 5.6	< 0.001	1,2 > 3
Inferior	53.3 ± 4.9	53.6 ± 4.3	51.5 ± 6.1	< 0.001	1,2 > 3
RNFL thickness (µm)					
Superior	102.4 ± 9.4	102.1 ± 9.9	97.5 ± 8.9	< 0.001	1,2 > 3
Inferior	99.0 ± 9.1	98.2 ± 7.9	93.1 ± 9.2	< 0.001	1,2 > 3
GCC thickness (µm)					
Superior	96.1 ± 6.1	96.5 ± 5.6	94.9 ± 5.5	0.029	2 > 3
Inferior	95.9 ± 6.1	96.3 ± 5.2	93.5 ± 5.6	< 0.001	1,2 > 3

AL = axial length; VD = vessel density; RNFL = retinal nerve fiber layer; GCC = ganglion cell complex; Tukey's test revealed statistically significant differences in means when comparing groups, with the symbols ' > ' and ' < ' indicating higher or lower means, respectively, in one group compared to another. On the other hand, the symbol ', ' signifies that there were no statistically significant differences between the groups being compared

**Table 5:** Pearson's Correlation Test between age with OCTA parameters and between Axial Length with OCTA parameters

	Age			AL	
	R	*p* value		r	*p* value
Macular VD					
Superior	−0.317	< 0.001		−0.022	0.633
Center	−0.302	< 0.001		0.357	< 0.001
Inferior	−0.244	< 0.001		−0.185	< 0.001
Peripapillary VD					
Superior	−0.171	< 0.001		−0.241	< 0.001
Inferior	−0.098	0.029		−0.227	< 0.001
RNFL Thickness					
Superior	−0.095	0.033		−0.207	< 0.001
Inferior	−0.071	0.115		−0.270	< 0.001
GCC Thickness					
Superior	−0.090	0.045		−0.061	0.179
Inferior	−0.099	0.028		−0.161	0.001

AL = axial length; VD = vessel density; RNFL = retinal nerve fiber layer; GCC = ganglion cell complex

**Table 6:** Multivariable linear regression models with generalized estimating equation (GEE) for correlations between age with OCTA parameters and between axial length with OCTA parameters

	Age				AL		
	*β*	SE	*p* value		*β*	SE	*p* value
Macular VD							
Superior	−0.106	0.019	< 0.001		−0.060	0.245	0.807
Center	−0.136	0.028	< 0.001		1.155	0.216	< 0.001
Inferior	−0.084	0.019	< 0.001		−0.522	0.205	0.011
Peripapillary VD							
Superior	−0.066	0.020	< 0.001		−0.733	0.215	0.001
Inferior	−0.042	0.023	0.066		−0.664	0.231	0.004
RNFL Thickness							
Superior	−0.064	0.042	0.130		−1.273	0.329	< 0.001
Inferior	−0.047	0.039	0.230		−1.356	0.308	< 0.001
GCC Thickness							
Superior	−0.033	0.027	0.214		−0.310	0.205	0.130
Inferior	−0.039	0.026	0.125		−0.599	0.208	0.004

VD = vessel density; RNFL = retinal nerve fiber layer; GCC = ganglion cell complex

**Fig. 2:**
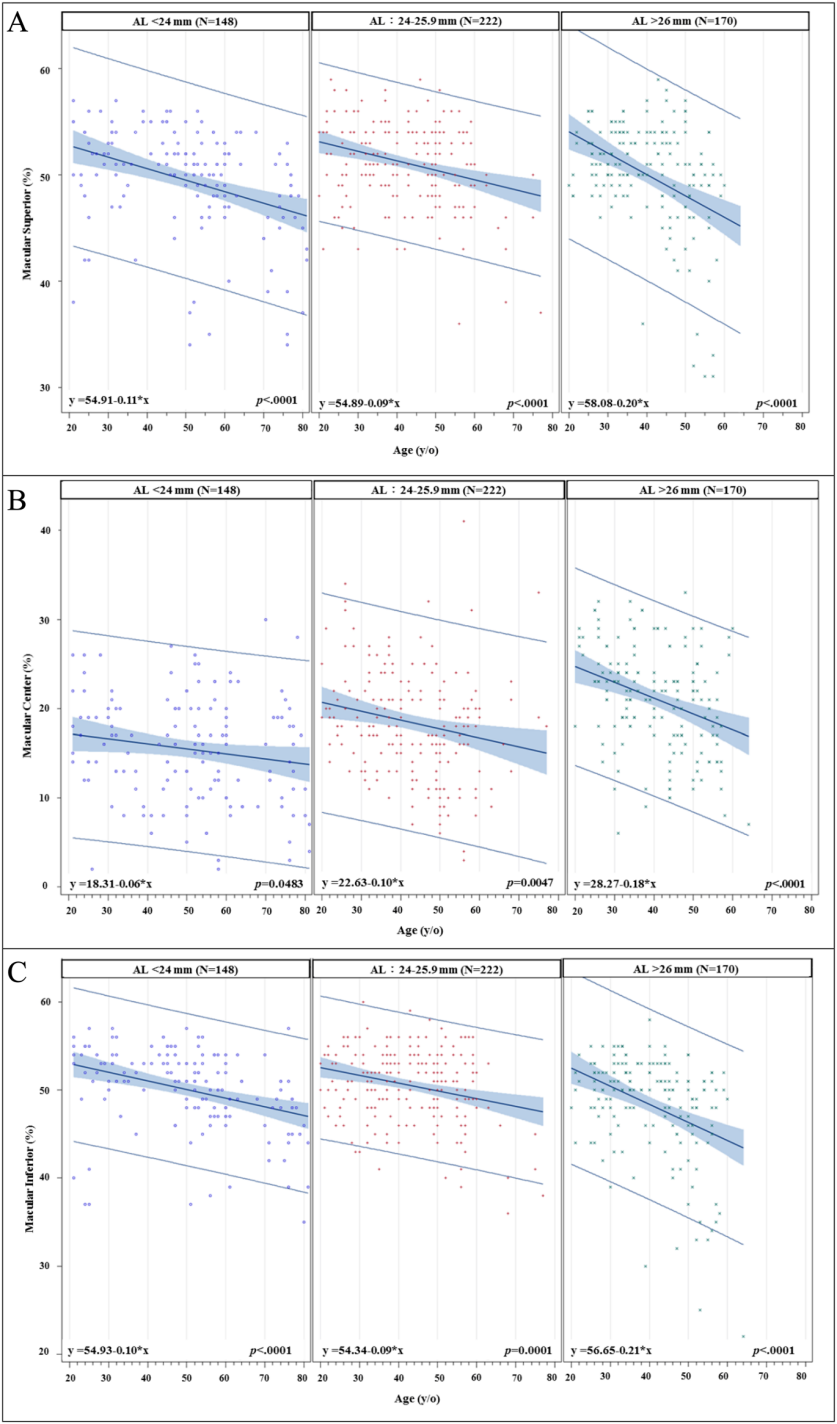
The rates of age-related macular SVD reduction in different axial length groups. Linear model of relationship between AL and the rates of age-related SVD reduction. (A) Macular Superior (B) Macular Center (C) Macular Inferior AL = axial length; SVD = superficial vessel density

**Fig. 3:**
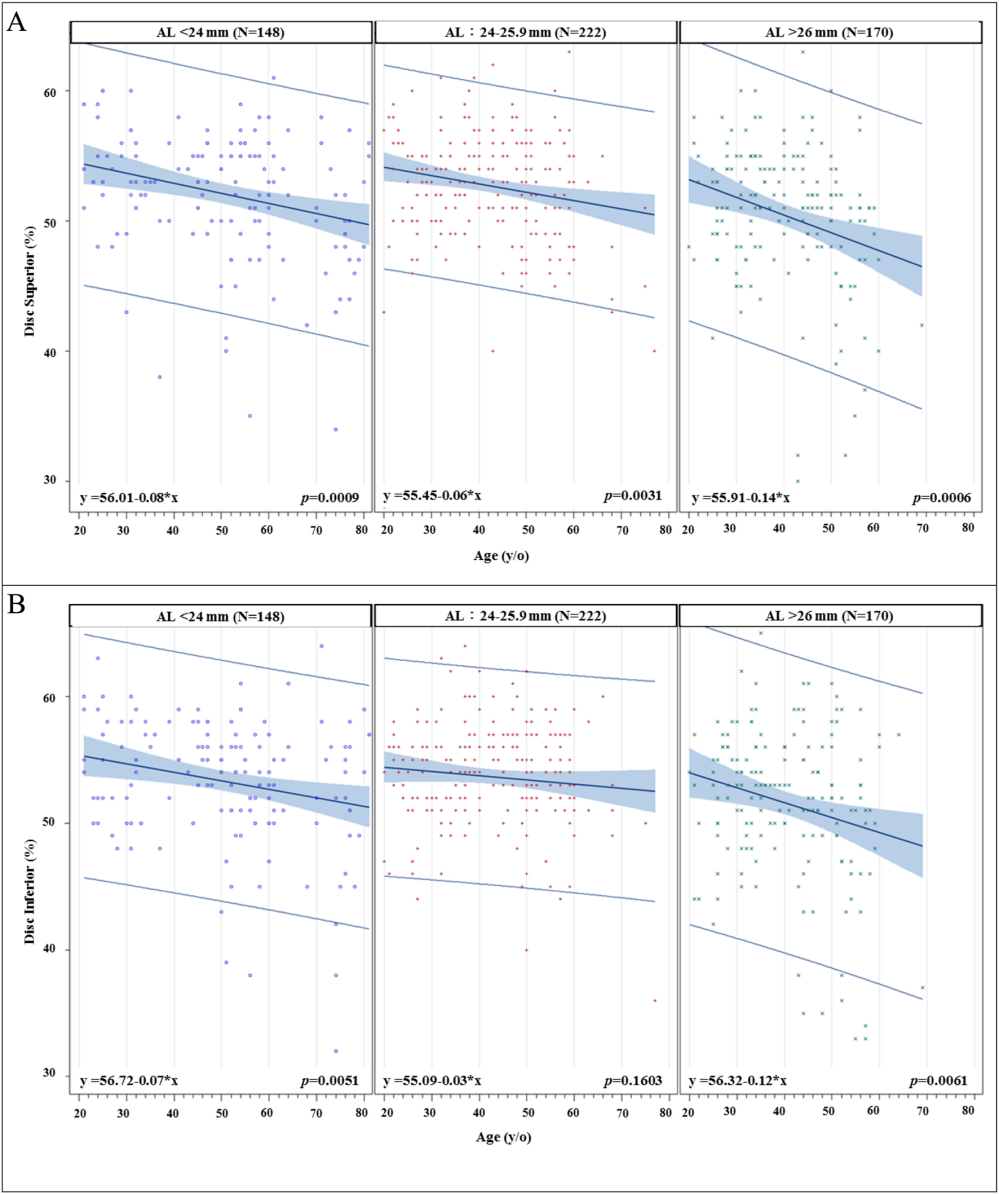
The rates of age-related peripapillary SVD reduction in different axial length groups. Linear model of relationship between AL and the rates of age-related SVD reduction. (A) Peripapillary Superior (B) Peripapillary Inferior AL = axial length; SVD = superficial vessel density

**Fig. 4:**
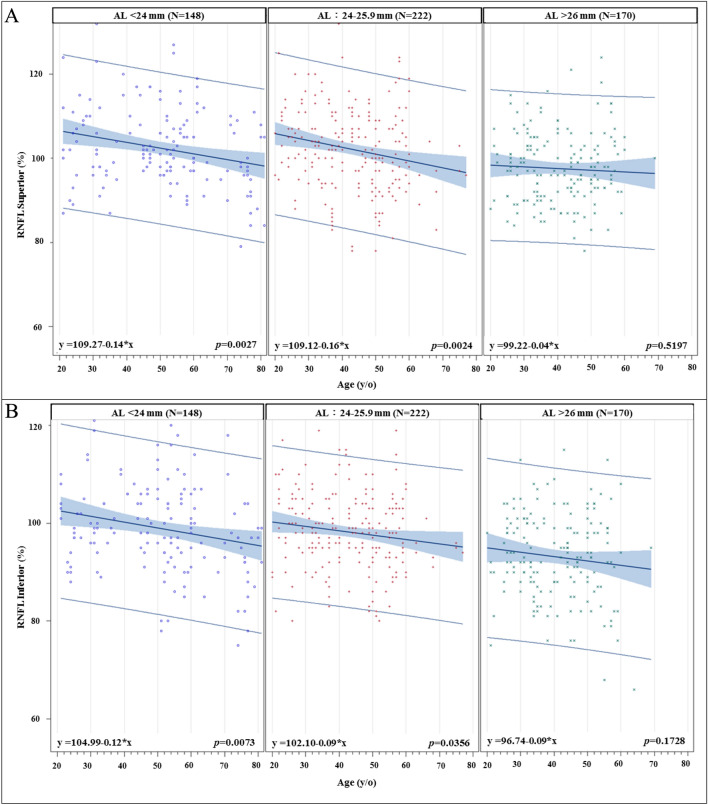
The rates of age-related RNFL thinning in different axial length groups. Linear model of relationship between AL and the rates of age-related RNFL thinning. (A) RNFL Superior (B) RNFL Inferior AL = axial length; RNFL = retina nerve fiber layer

**Fig. 5:**
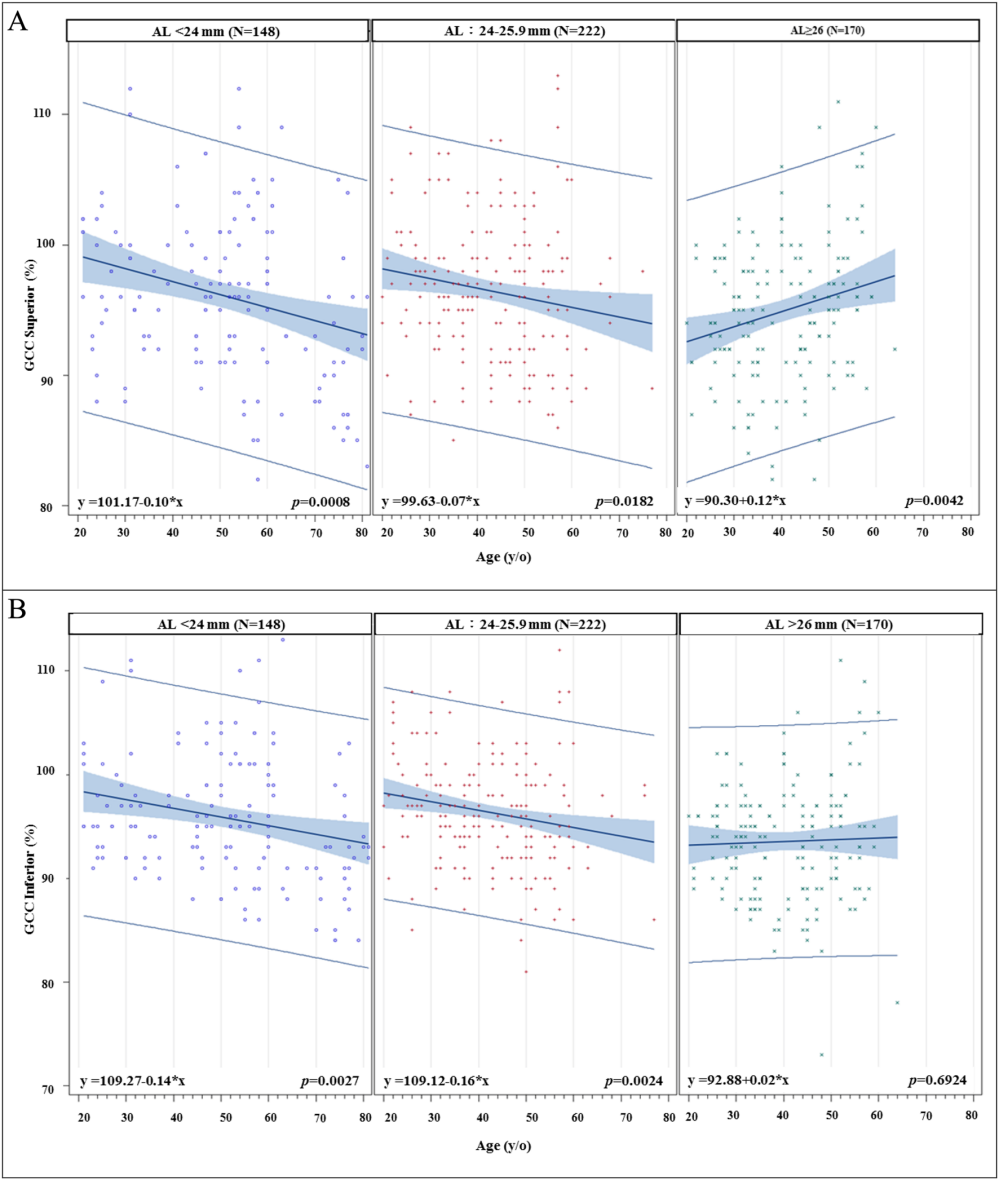
The rates of age-related GCC thinning in different axial length groups. Linear model of relationship between AL and the rates of age-related GCC thinning. (A) GCC Superior (B) GCC Inferior AL = axial length; GCC = ganglion cell complex

We further divided our study population into three different groups based on axial length: short (≤ 24 mm), medium (24 to 26 mm), and long (> 26 mm) AL groups. The slopes of age-related superior, center, and inferior macular SVD reduction are shown in Fig. 2. The steepest macular SVD reduction slopes are all from the long AL group in the macular superior, center, inferior regions (all *p* < 0.001). For the age-related superior and inferior peripapillary SVD reduction rates (Fig. 3), it also shows that the steepest peripapillary SVD reduction slopes are from the long AL group in the peripapillary superior and inferior regions (all *p* < 0.01). The age-related thinning rates of RNFL thickness (Figs. 4 and 5), no significant relationships were found in the long AL group for thickness measurements except for superior GCC thickness.

## Discussion

Due to the high prevalence of myopia and glaucoma in Asia, especially in Taiwan, the effect of myopia on OCTA needs to be clarified. Moreover, the normative database of SVD data in Asians is drawn from only a few studies [7, 23]. Only when we achieve enough reference data can we confidently utilize OCTA in clinical practices such as early detection of glaucoma or even long-term follow-ups.

In the present study, our main findings included mean SVD in the superior, central, and inferior macula and the superior and inferior peripapillary area, which were 50.3 ± 4.8%, 18.4 ± 6.4%, 49.7 ± 5.0%, 51.8 ± 4.9%, 52.9 ± 5.2%, respectively. SVD in all areas except central macula showed a negative correlation between age and AL. In a large population-based study in Hong Kong (You et al.) [7] using a spectral-domain optical coherence tomography (SD-OCT) device (Optovue, Inc., Fremont, CA, USA), it was reported that the mean SVD in the superior and inferior macular area were 49.69% and 48.41%, respectively, similar to our data. Decreasing SVD levels with older subjects and longer ALs were also found in the same study (Kiziltunc et al.) [24] using the same OCTA device by Optovue, Inc. on 80 healthy eyes of Turkish subjects, showing a mean macular parafoveal SVD of 53.2% and a mean peripapillary SVD of 53.0%. The higher values they obtained are likely attributable to the different ethnicity of the subjects and the fact that our study included many more myopic participants from a technologically well-developed city. Another study by Fernandez-Vigo et al. examined 346 healthy subjects by a swept-source OCTA (Topcon, Tokyo, Japan) device and observed SVD in peripapillary superior and inferior areas of 59.9% and 62.1%, respectively. Although the SVD values were higher than what we observed, their study found the peripapillary inferior area SVD to be higher than the peripapillary superior area SVD, as our study also found. The higher SVD may be explained by the younger subjects (the mean age was 37.7 ± 19.8 years) enrolled in that study and the use of a larger scan area (6 × 6 mm^2^). Additionally, previous studies revealed that SVD values measured using different algorithms and OCTA devices were not interchangeable [25, 26].

We found that macular SVD was negatively correlated with age, which most studies have corroborated [7, 23, 27–31]. You et al. [7] reported that macular SVD was stable or even slightly increased with age until age 50, at which point it started to decrease progressively at a rate of approximately 1.3% per decade. Hashmani et al. [30] found a significant decrease in SVD after the fifth decade of life. According to multivariable linear regression models with GEE in the current study, we also demonstrated that SVD of the superior, central and inferior macular areas decreased at rates of 1.06%, 1.36%, and 0.84%, respectively, every decade. Jo et al. [31] reported that perifoveal SVD significantly decreased by −0.059%/year in the superior area, while the inferior area showed a nonsignificant decrease of −0.031%/year. Interestingly, we also found the correlation between age and inferior peripapillary SVD to be weaker than that with the superior quadrant using both Pearson’s correlation (*p* = 0.029) and multivariable linear regression models with GEE (*p* = 0.066). In the same way, the inferior RNFL thickness showed a pattern similar to the inferior peripapillary SVD in the analysis. The correlation between age and inferior RNFL thickness was also weaker than that with the superior quadrant in both Pearson’s correlation test (*p* = 0.115) and multivariable linear regression models with GEE (*p* = 0.230). Further studies should be performed in the future to discuss the reason why the inferior correlation was lower than the superior part. In contrast, Rao et al. [32] found no correlations between age and peripapillary SVD measurements in a healthy Indian population (*n* = 46). Bazvand et al. [33] also reported no significant differences in peripapillary SVD across different age groups (ranging from 12 to 67 years; *n* = 79). The different results might come from smaller sample sizes and younger subjects in their studies or different races.

Regarding the correlation between AL and SVD, some OCTA studies on highly myopic participants, with a refractive error greater than –6 diopters or AL longer than 26 mm [18–20], demonstrated longer AL accompanied by decreased superficial vessel density measurements. Min et al. [19] showed that a long AL (> 26 mm) had an enlarged foveal avascular zone (FAZ) area and a reduced SVD before the occurrence of the pathological changes associated with myopia. Leng et al. [18] compared the macular superficial capillary plexus (SCP), another parameter to interpret SVD, between age-matched groups of eyes with longer ALs (AL > 26 mm) and eyes with shorter ALs (AL < 23 mm). The results indicated that SCP was significantly lower in longer AL eyes, which is comparable to our data. We further analyzed different sectors in the macula. Nevertheless, superior macular SVD and GCC thickness showed no correlations with AL change in Pearson’s correlation and multivariable linear regression with GEE. The reason for this finding was unclear and should be confirmed in future studies. Notably, the long AL group showed the steepest age-related SVD reduction rates in the macula and the peripapillary area. On the other hand, the age-related thinning rates of RNFL and GCC thickness in the long AL groups were mostly not significant. This illustrated that the AL had an apparent effect on SVD changes, especially in populations with high myopia, while it had less of an effect on thickness changes.

It is commonly believed that high myopia is associated with elongation of the eyeball. The blood vessels supplying the disk could possibly adjust to the longer shape and eventually become damaged, resulting in decreased blood perfusion [20]. In other words, vascular changes by AL were more apparent than those by age in most retinas. In our GEE models, SVD in the inferior macula and superior and inferior peripapillary areas was −0.522%/mm, −0.733%/mm, and −0.664%/mm, respectively. Min et al. [19] reported an r value of −0.340 for the association between axial length and macular SVD, and Guo et al. [20] reported a *β* value of −0.479 in their multivariable regression analysis of effect of axial length on average superficial parapapillary microvascular density. The lower β value they obtained was due to the younger subjects (the mean age was 21.93 ± 2.93 years) they enrolled in the study and the smaller sample size (*n* = 174).

Many researchers have used OCT parameters to evaluate retinal structural changes with age with OCT parameters. Wei et al. [34] showed that age has a significantly negative correlation with inner retinal layer thickness. In contrast, Schuster et al. [35] found no significant correlation between age and RNFL thickness while highlighting the importance of refraction when evaluating RNFL thickness. They utilized models with GEE based on data from 306 healthy Caucasian subjects to evaluate correlations between peripapillary RNFL thickness and other factors. Our past study [36] reported superior RNFL thickness in bilateral eyes with a significant thinning rate of 0.7 μm/year (by linear regression analysis). This result was not completely consistent with our current study. Per the Pearson’s correlation test shown in Table 5, superior RNFL thickness was negatively correlated with age (*p* = 0.033). When we delved deeper with multivariable linear regression models with GEE, age still had a negative, albeit nonsignificant, correlation with superior RNFL thickness (*p* = 0.130). This inconsistency is believed to be due to the use of different statistical methods. However, it is believed that multivariable linear regression models with GEE will be more accurate for this type of statistical analysis.

Age and AL were the major factors affecting SVD, RNFL thickness and GCC thickness. The effects of each factor can be interpreted in Table 6. The effect of age on RNFL thickness and GCC thickness in all regions was not significant in the present study, but there was a clearly significant on SVD. In a recent longitudinal study by Hou et al., the normalized change rate of perifoveal SVD was −1.62%/year, while the GCC thickness was -1.34%/year in their healthy controls. In other words, age has a greater effect on vascular changes than on structural changes. Turning to the effect of AL, AL was significantly associated with all the dependent variables aside from superior macular SVD and superior GCC thickness. We compared the AL effect on inferior GCC thickness with inferior macular SVD, superior RNFL thickness with superior peripapillary SVD, and inferior RNFL thickness with inferior peripapillary SVD. The *β*-value revealed that the effect of AL on structural changes (thickness) had a steeper slope than the effect of AL on vascular changes (SVD). An assumption can be made that AL has a greater effect than age and is more significant on thickness changes than SVD changes. However, it was unclear why central macular SVD was positively correlated with longer AL in the present study. This may be due to the lack of blood supply in the fovea as a result of its anatomy.

In addition to age and AL, the importance of systemic factors such as HDL, estimated glomerular filtration rate (eGFR) and hemodynamic parameters (MAP, HR) on SVD should not be ignored. To the best of our knowledge, there are no studies on SVD with each specific systemic factor in healthy subjects. Although we found no hemodynamic parameters (MAP, HR) that were significant factors in macular or peripapillary SVD, a study in 2019 by Muller et al. [37] on diurnal changes in forty primary open-angle glaucoma patients reported that macular flow density, another OCTA parameter similar to SVD, was influenced significantly by MAP and HR, while flow density in the peripapillary area was influenced significantly by HR only. More studies are needed to confirm the exact relationship.

The present study has certain limitations. First, due to the nature of the cross-sectional design, we could not definitively identify the mechanism. Future longitudinal studies are expected. Second, all of the enrolled participants were from our clinic. Selection bias in our population was inevitable. Participants over 50 years old were generally much less myopic and had shorter axial lengths than the younger participants in our study population. Although GEE adjusted for those interactions, the precise influences remained unclear.

## Conclusions

In conclusion, SVD measurements in the macula and peripapillary areas showed variation in non-glaucomatous individuals. Age and AL were the two main factors affecting SVD changes. AL was found to have a greater effect than age and showed a more significant effect on thickness than SVD. The steepest age-related SVD reduction rates in the macula and peripapillary areas were all in the long AL group. In short, the effects of AL on SVD should be kept in mind during the clinical application of OCTA, especially in areas with a high prevalence of myopia.
